# Fermented *Astragalus* in diet improved laying performance, egg quality, antioxidant and immunological status and intestinal microbiota in laying hens

**DOI:** 10.1186/s13568-020-01092-6

**Published:** 2020-08-31

**Authors:** Hong-Tao Shi, Bai-Yu Wang, Chuan-Zhou Bian, Ying-Qian Han, Hong-Xing Qiao

**Affiliations:** 1 College of Veterinary Medicine, Henan University of Animal Husbandry and Economy, Zhengzhou, 450046 Henan China; 2 Key Lab of Freshwater Animal Breeding, Key Laboratory of Agricultural Animal Genetics, Breeding and Reproduction, Ministry of Education, College of Fishery, Huazhong Agricultural University, Wuhan, 430070 Hubei China; 3 College of Animal Sciences and Veterinary Medicine, Henan Agricultural University, Zhengzhou, 450046 Henan China

**Keywords:** Fermented *astragalus*, Laying hens, Egg quality, Antioxidant and immunological status, 16S rRNA, Intestinal microbiota

## Abstract

In the era of increased antibiotic resistance and ever-stricter control on antibiotic use, it is urgent to develop green, safe, and non-residue alternatives to antibiotics applied to the poultry industry. To this end, we supplied the potential *Lactobacillus plantarum* (*L. plantarum*) fermented *Astragalus* in the diet of laying hens, with a final addition of 3‰. Its effects have been assessed on laying performance, egg quality, antioxidant and immunological status, and intestinal microbiota, and are compared to the control group, to the *Astragalus* group containing 3‰ unfermented *Astragalus*, and to the *L. plantarum* group containing 2% *L. plantarum* [5 × 10^8^ colony-forming unit (CFU) per milliliter (mL)]. During the second half of the experimental period (15 to 28 days), the egg production rate was considerably higher in the fermented *Astragalus* group than that in the other groups, with the fermented *Astragalus* group having the lowest feed conversion ratio. No significant difference (*P* > 0.05) was noted among treatments on egg quality. Fermented *Astragalus*-treated hens exhibited significantly increased catalase (CAT), glutathione peroxidase (GSH-Px), superoxide dismutase (SOD) and total antioxidant capacity (T-AOC) in serum, and reduced malondialdehyde (MDA) in serum. Furthermore, fermented *Astragalus* supplementation resulted in a significant increase in ileal microbiota abundance relative to control. In conclusion, feeding laying hens with *L. plantarum* fermented *Astragalus* has beneficial effects on production, antioxidant potential, immunity, and ileal microbiota. *L. plantarum* fermented *Astragalus* is expected to be a novel feed additive used in poultry production.

## Introduction

Eggs are one of the most crucial sources of animal protein and nutritional content in human diets. Due to the widespread use of antibiotics in poultry, drug residues in eggs have been gaining worldwide concern over the past few years (Vandemaele et al. 2002). In addition, antibiotic abuse has led to intestinal dysbacteriosis, diarrhea, immunocompromised state (Willing et al. 2011). Thus, it is imperative to develop green, safe, and non-residue alternatives to antibiotics applied to the poultry industry. Traditional Chinese herbal medicines are the gem of China with characteristics of safety, efficiency, and low residue and are commonly used in preventive or therapeutic strategies for animal diseases (Xu et al. 2017). Traditional Chinese herbal medicines have utilised as feed additives for growth promotion and improvement of immunity and various effects, including anti-bacterial, anti-viral and antioxidative activities (Patra et al. 2015; Wang et al. 2015). Since ancient times, traditional Chinese herbal medicines can be processed by microbial fermentation for improving its quality (Zhu et al. 2014). For example, fermentation of Chinese herbal medicine mediated by microbes can degrade macromolecule-materials into small ones and reduce their side effects (Ai et al. 2019). Because microorganisms and their metabolic products can regulate the bioactive products of traditional Chinese herbal medicines, there is a close relationship between microorganisms and traditional Chinese herbal medicines.

*Astragalus* is a universal traditional Chinese herbal medicine and its main active pharmaceutical ingredients include polysaccharides, saponins, flavonoids, anthraquinones, alkaloids, amino acids, β-sitosterol and metallic elements (Li et al. 2014). *Astragalus* has been reported to possess anti-inflammatory (Kim et al. 2013), anti-viral (Kallon et al. 2013) and antioxidant (Shahzad et al. 2016) activities and to enhance immunity (Qin et al. 2012), and it has been widely used in livestock. Nevertheless, challenges to the extraction yield of *Astragalus* functional ingredients are raised due to the recalcitrance of plant cell walls, and novel strategies for the improvement of *Astragalus* utilization efficiency have to be focused. The trend of microbial fermentation offers the possibility of addressing the above problem. In recent years, research revealed that utilizing the fungus *Aspergillus* to ferment the *Astragalus* can significantly increase its phenolic contents and antioxidant activity, and the solid-state bio-processing strategy could be an innovative approach to enhance the antioxidant activity of *Astragalus* (Sheih et al. 2011). Our previous studies have verified that the solid fermentation of *Astragalus* by *L. plantarum* promotes the extraction yield of *Astragalus* active components and the production yield of organic acids (Qiao et al. 2018c). Further investigation showed that fermented *Astragalus* improves broiler growth performance, enhances serum antioxidant status, and reduces fecal pathogenic microbiota of broiler chickens (Qiao et al. 2018b).

Over the last few years, there has meant considerable research on the application of *Astragalus* polysaccharide as a feed additive in livestock including laying hens. However, there has not been a systematic appraisal of the application of *Astragalus* fermented by *L. plantarum* as a feed additive for laying hens. In this study, we investigated the possible effects of *Astragalus* fermented by *L. plantarum* on egg production, egg quality, antioxidant status, immune factors expression and gut microbiome of laying hens, combining the classical culture and detection methods with high throughput sequencing.

## Materials and methods

### Fermentation of Astragalus

*Lactobacillus plantarum* (CGMCC 1.557) was purchased from the China General Microbiological Culture Collection Center (CGMCC) (Beijing, China). Te dried root of *Astragalus membranaceus* (Fisch.) Bge. var. mongholicus was obtained from Gansu Huisen Pharmaceutical Development Co., Ltd. (Minxian, Gansu, China) and verified by Dr. JingYu Zhang (Henan University of Traditional Chinese Medicine, Zhengzhou, Henan, China). The purchased *Astragalus* was crushed into powder and filtered with a 100-mesh filter for further studies. The fermentation of *Astragalus* was performed following the method reported in our previous publications with slight modification (Qiao et al. 2018b). Briefly, dried *Astragalus* powder (7500 g) was inoculated with *L. plantarum* (1 × 10^6^ CFU per gram) with a water content of 45% and *Astragalus*–*L. plantarum* mixtures were divided equally into 35 × 45-mm plastic film bags. The bags were sealed for fermentation at 37 °C for 30 days and then dried out at room temperature for future use.

### Experimental design, diets and management

Two hundred and forty healthy Hy-Line Gray hens (351 days, Zhengzhou, China) were acclimated with the basal diets for 7 days. Then, hens were randomly divided into four groups (fermented *Astragalus* group, *Astragalus* group, *L. plantarum* group, and control group), each containing five replicates, with 12 hens per replicate. The control group was fed with the basal diet; the *L. plantarum* group was fed with the basal diet supplemented with 2% *Lactobacillus* solution (5 × 10^8^ CFU/mL) through uniform spraying; the *Astragalus* group was fed with the basal diet supplemented with 3‰ *Astragalus*, and fermented *Astragalus* group was fed with the basal diet supplemented with 3‰ fermented *Astragalus* (pre-experimental results showed that supplementing at a rate of 3‰ of diet achieves optimal results). The trial lasted for 35 days (7-day adaptation period and 28-day experimental stage). The hens were housed in a clean environment with good ventilation and artificial lighting allowed 16 h of lighting per day, and with water and food ad libitum. The basal diet of all groups was the same and prepared according to the NRC (1994) laying hen nutrition requirement standard. The composition and nutrient levels of the basal diet were shown in Table 1. All animal experiments were conducted according to the Guidelines for the Care and Use of Experimental Animals established and approved by the Laboratory Animal Management Committee of Henan University of Animal Husbandry and Economy (HNMY 1606).

**Table 1:** Composition and nutrient levels of the basal diet (air-dry basis %)

Ingredients	Content	Nutrient levels	Content
Corn	61.4	ME/(MJ/kg)^b^	11.01
Soybean meal	23.8	CP	15.49
Wheat bran	2.0	Ca	3.50
Soybean oil	0.6	TP	0.56
CaHPO_4_	1.3	AP	0.35
Limestone	8.6	Lys	0.74
NaCl	0.3	Met + Cys	0.51
Premix^a^	2.0		
Total	100.0		

^a^ The premix provided the following per kilogram of the diet: VA 11,000 IU, VD_3_ 3200 IU, VE 25 IU, VK_3_ 2.2 mg, VB_1_ 1.5 mg, VB_2_ 3.5 mg, VB_12_ 3 mg, nicotinic 28 mg, calcium pantothenate 8.5 mg, biotin 0.5 mg, choline 255 mg, Fe 55 mg, Zn 62 mg, Cu 6 mg, Se 0.20 mg

^b^ ME was a calculated value, while the others were measured values

### Hen productivity and egg quality

During the experimental period, egg production, broken egg production, egg weight, and feed intake were recorded daily. The egg production rate and the feed conversion ratio (FCR) (feed intake/egg weight gain) during day 1 to day 14 and day 15 to day 28 were calculated to assess the laying performance. On day 14 and day 28, five eggs from each replicate were randomly sampled and measured egg quality parameters of egg shape index (ESI), eggshell strength (ESS), eggshell thickness (EST), albumen height (AH), Haugh unit (HU), yolk color (YC) and yolk weight (YW).

### Serum antioxidant indices

On day 14 and day 28, one hen from each replicate was randomly selected. Following blood collection from the heart, the serum was isolated and stored at − 20 °C until use. The CAT assay kit (Catalog number R22072), GSH-Px assay kit (Catalog number R21876), SOD assay kit (Catalog number R22262), T-AOC assay kit (Catalog number R24147) and MDA assay kit (Catalog number R21869) were purchased from Shanghai yuanye Bio-Technology Co., Ltd (Shanghai, China).

### Real-time qPCR

After blood samples collection, liver, spleen, ileum, and cecum samples were harvested for interferon-gamma (IFN-γ) and tumor necrosis factor-alpha (TNF-α) mRNA expression evaluation. Total RNA was extracted from these tissues using RNAiso Plus (Catalog number 9108, Takara, Otsu, Shiga, Japan) and reverse transcribed into cDNA with PrimeScript™ RT reagent Kit with gDNA Eraser (Perfect Real Time) (Catalog number RR047, Takara, Otsu, Shiga, Japan) according to the manufacturer’s protocol. The primers used in the study were synthesized by Sangon Biotech (Shanghai) Co., Ltd. (Shanghai, China), and primer sequences are summarized in Table 2. The Real-time qPCR reactions were performed using a TB Green Premix EX Taq (Catalog number RR420, Takara, Otsu, Shiga, Japan) in a 7500 Fast Real-Time PCR System (Thermo Fisher). β-actin was used as a housekeeping gene. The relative mRNA expression levels of the target genes compared to the housekeeping gene were calculated using the 2^−ΔΔCt^ method.

**Table 2:** Primers used for quantitative real-time PCR analysis

Primers	Sequences 5′ ~ 3′	Size
IFN-γ-F	AACAACCTTCCTGATGGCGT	107 bp
IFN-γ-R	TGAAGAGTTCATTCGCGGCT	
β-actin-F	TATGTGCAAGGCCGGTTTCG	170 bp
β-actin-R	CAATGGGGTACTTCAGGGTCAG	
TNF-α-F	GCCCTTCCTGTAACCAGATG	71 bp
TNF-α-R	ACACGACAGCCAAGTCAACG	

### Sample collection and DNA extraction

On day 14 and day 28, a total of 48 hens were randomly selected (12 hens per group) and euthanized via an intravenous injection of pentobarbital sodium (150 mg/kg) to collect ileal and cecal contents (Pan et al. 2018). The samples were named as the 14-d ileum control group (14IA), 14-d ileum *Astragalus* group (14IB), 14-d ileum *L. plantarum* group (14IC), 14-d ileum fermented *Astragalus* group (14ID), 14-d cecum control group (14CA), 14-d cecum *Astragalus* group (14CB), 14-d cecum *L. plantarum* group (14CC), 14-d cecum fermented *Astragalus* group (14CD), 28-d ileum control group (28IA), 28-d ileum *Astragalus* group (28IB), 28-d ileum *L. plantarum* group (28IC), 28-d ileum fermented *Astragalus* group (28ID), 28-d cecum control group (28CA), 28-d cecum *Astragalus* group (28CB), 28-d cecum *L. plantarum* group (28CC), and 28-d cecum fermented *Astragalus* group (28CD). All collected samples were immediately stored at – 20 °C until extraction. DNA extraction was performed with a commercial DNA extraction kit (Tiangen Biotech Corporation, Beijing, China) and quantified by a Qubit 2.0 fluorometer (Invitrogen Corporation, Carlsbad, CA, USA). The extracted DNA was qualitatively assessed by 0.8% agarose gel electrophoresis and spectrophotometry (optical density at 260/280 nm) and stored at − 20 °C until further analysis.

### 16S rRNA gene sequencing and analysis

For amplicon library generation, the V4 region of the 16S rRNA gene of all DNA samples was amplified with gene-specific primers (F: 5′- AYTGGGYDTAAAGNG-3′; R: 5′-TACNVGGGTATCTAATCC-3′). PCR amplifications were performed using Q5 high-fidelity PCR DNA polymerase (Catalog number M0491, New England Biolabs, Ipswich, MA, USA) and completed under the following conditions: a pre-denaturation at 98 °C for 30 s; 27 cycles of 98 °C for 15 s, 50 °C for 30 s, and 72 °C for 30 s; a final extension at 72 °C for 5 min. Amplicons were purified using the Axygen AxyPrep DNA Gel Extraction Kit (Catalog number AP-GX-250G, Corning Life Sciences, Corning, NY, USA). DNA libraries were validated and quantified using the TruSeq Nano DNA LT Library Preparation Kit (Catalog number FC-121-4001, Illumina, San Diego, CA, USA) and Quant-iT™ PicoGreen™ dsDNA Assay Kit (Catalog number P11496, Invitrogen Corporation, Carlsbad, CA, USA). After quantification, the barcoded V4 amplicons were pooled to a final concentration of 2 nmol/L and sequenced using an Illumina MiSeq platform to generate 300 bp paired-end reads. Raw reads were quality-filtered to remove any reads less than 150 bp using Quantitative Insights into Microbial Ecology (QIIME) version 1.8 (Caporaso et al. 2010) and clustered into Operational Taxonomic Units (OTUs) based on a 97% similarity threshold. The representative sequence was chosen based on the abundance and was aligned under a given taxonomic classification using the Greengenes database, and low abundance OTUs of archaea and eukaryotes were removed (Bokulich et al. 2013). Alpha-diversity was calculated with Chao1 and ACE estimators, Shannon and Simpson indices. Partial least squares discriminant analysis (PLS-DA) was performed using QIIME software package v1.8 to discriminate between different groups (day 14 and day 28) and to establish β-diversity. The sequences generated in this study have been deposited in the National Center for Biotechnology Information sequence read archive (https://www.ncbi.nlm.nih.gov/biosample) under the Accession number SRA: PRJNA533918.

### Statistical analysis

Only for genes mRNA expression assay, statistical analyses were performed with Student’s t-test and graphed using GraphPad Prism 6.00 (GraphPad Software), and significance levels are indicated as *P < 0.05, **P < 0.01, ***P < 0.001, ****P < 0.0001. All other statistical analyses were performed by one-way analysis of variance using SPSS 24.0 software, and all data were expressed as means ± SD, with *P* < 0.05 considered statistically significant.

## Results

### Hen productivity and egg quality

The effects of different dietary supplements on the laying hen production performance and egg quality are listed in Tables 3 and 4. During day 1 to day 14, there were no differences in the laying rate and FCR among four groups (*P* > 0.05), with hens fed with fermented *Astragalus* had the highest laying rate. During day 15 to day 28, hens fed with fermented *Astragalus* had the highest laying rate, 7.14% higher than that of the control group (*P* < 0.05). Although the differences were not statistically significant (*P* > 0.05), the laying rate of the *Astragalus* group and *L. plantarum* group were also increased by 3.25% and 2.99%, respectively in comparison with the control group. Furthermore, the FCR of the fermented *Astragalus* group was reduced by 6.6% compared with that of the control group (*P* < 0.05), while the FCR of the *Astragalus* group and *L. plantarum* group displayed no significant differences as compared with the controls (*P* > 0.05). In addition, no significant differences in the phenotype of the egg quality including ESI, ESS, EST, AH, YC, HU, and YW were observed among dietary treatments, suggesting that dietary supplements have no significant effects on egg quality in this study. Therefore, we identified that dietary supplementation of fermented *Astragalus* can markedly improve egg production and decrease FCR, and the effect is substantially superior to that of *Astragalus* and *L. plantarum*.

**Table 3:** Effects of different dietary supplements on the production performance of laying hens

Parameters	Control	*Astragalus*	*L. plantarum*	Fermented * Astragalus*	*P-*value	SEM
1–14 days						
Laying rate, %	87.50 ± 3.45	89.17 ± 2.08	87.50 ± 2.70	90.60 ± 3.80	0.349	1.376
FCR	2.02 ± 0.08	1.94 ± 0.05	2.02 ± 0.10	1.95 ± 0.08	0.224	0.037
15–28 days						
Laying rate, %	88.96 ± 3.76a	92.21 ± 2.75ab	91.95 ± 3.83ab	96.10 ± 1.03b	0.017	1.368
FCR	1.81 ± 0.08b	1.75 ± 0.06ab	1.77 ± 0.11ab	1.69 ± 0.05a	0.172	0.035

Different lowercase letters in the same row indicate significant difference (*P* < 0.05), and the same letters or no letters indicate no significant difference (*P* > 0.05) (same as below)

*FCR* feed conversion ratio

**Table 4:** Effects of different dietary supplements on the egg quality

Parameters	Control	*Astragalus*	*L. plantarum*	Fermented * Astragalus*	*P-*value	SEM
14 days						
ESI	1.29 ± 0.02	1.29 ± 0.03	1.29 ± 0.03	1.29 ± 0.03	0.961	0.012
ESS (kg/N)	3.59 ± 0.41	4.04 ± 0.33	3.87 ± 0.32	3.90 ± 0.40	0.302	0.164
EST (mm)	0.344 ± 0.013	0.344 ± 0.011	0.346 ± 0.008	0.348 ± 0.008	0.927	0.005
AH (mm)	8.74 ± 0.43ab	8.74 ± 0.63ab	8.47 ± 0.51a	9.21 ± 0.33b	0.162	0.219
YC	5.15 ± 0.32	5.00 ± 0.28	5.44 ± 0.21	5.32 ± 0.40	0.160	0.139
HU	92.84 ± 1.90	92.16 ± 3.42	92.10 ± 2.82	94.75 ± 1.61	0.183	1.138
YW	15.75 ± 0.37	15.47 ± 0.32	15.60 ± 0.22	15.27 ± 0.73	0.417	0.202
28 days						
ESI	1.28 ± 0.02	1.28 ± 0.01	1.29 ± 0.01	1.29 ± 0.03	0.452	0.008
ESS (kg/N)	4.31 ± 0.37	4.34 ± 0.60	4.20 ± 0.16	4.54 ± 0.41	0.625	0.185
EST (mm)	0.375 ± 0.014	0.392 ± 0.020	0.389 ± 0.013	0.387 ± 0.018	0.396	0.007
AH (mm)	8.80 ± 0.40	8.92 ± 0.43	8.85 ± 0.24	9.08 ± 0.44	0.676	0.173
YC	5.59 ± 0.39	5.55 ± 0.47	5.22 ± 0.12	5.44 ± 0.45	0.435	0.170
HU	92.57 ± 2.19	92.80 ± 2.42	92.74 ± 1.03	93.80 ± 2.07	0.763	0.895
YW	15.40 ± 0.39	15.50 ± 0.59	15.78 ± 0.38	15.76 ± 0.58	0.556	0.222

*ESI* egg shape index, *ESS* eggshell strength, *EST* eggshell thickness, *AH* albumen height, *HU* haugh unit, *YC* yolk color, *YW* yolk weight

### Serum antioxidant indices

The effects of different dietary supplements on the laying hen antioxidant status are listed in Table 5. The data indicated that all dietary supplementation did not have an effect on the biomarkers of antioxidative stress at day 14 (*P* > 0.05). However, serum CAT, GSH-Px, SOD, and T-AOC concentrations were increased by 61.5%, 62.4%, 68.0%, and 52.6% (*P* < 0.05) at the end of experimentation in the fermented *Astragalus* group as compared with the controls. No statistically significant differences were observed for CAT, GSH-Px, and SOD among the control, *Astragalus*, and *L. plantarum* groups (*P* > 0.05). Among the effects of different dietary supplements on MDA activity in serum of laying hens, hens fed with fermented *Astragalus*, *Astragalus* diet were significantly decreased by 54.7% and 43.0% than that of the control treatment (*P* < 0.05); treatment with *L. plantarum* diet did not dramatically differ from the control treatment (*P* > 0.05). The results presented above show that dietary supplementation of fermented *Astragalus* can markedly improve laying hen antioxidant status, and the effect is significantly superior to that of *Astragalus* and *L. plantarum*.

**Table 5:** Effects of different dietary supplements on the antioxidant status of laying hens

Parameters	Control	*Astragalus*	*L. plantarum*	Fermented *Astragalus*	*P-*value	SEM
14 days						
CAT (U/mL)	43.90 ± 2.46	57.21 ± 13.66	76.80 ± 44.15	61.52 ± 31.03	0.445	13.960
GSH-Px (U/L)	90.80 ± 14.36	115.32 ± 32.20	145.76 ± 79.19	120.37 ± 54.47	0.450	23.989
SOD (U/mL)	276.62 ± 44.10	313.42 ± 87.16	321.99 ± 91.99	339.02 ± 127.71	0.757	43.954
T-AOC (U/mL)	12.25 ± 2.13	14.33 ± 4.66	15.40 ± 4.63	14.79 ± 6.31	0.753	2.211
MDA (nmol/mL)	6.93 ± 0.52	8.58 ± 2.50	8.71 ± 2.62	8.63 ± 3.52	0.734	1.276
28 days						
CAT (U/mL)	47.93 ± 2.41a	45.58 ± 8.17a	86.63 ± 17.73ab	124.58 ± 66.85b	0.020	17.563
GSH-Px (U/L)	90.07 ± 20.18a	108.12 ± 31.99a	159.86 ± 36.77ab	239.78 ± 142.58b	0.043	35.992
SOD (U/mL)	230.98 ± 29.88a	321.95 ± 125.37a	473.54 ± 125.13ab	722.33 ± 418.70b	0.030	108.041
T-AOC (U/mL)	10.78 ± 1.62a	12.81 ± 2.82a	20.29 ± 4.68b	22.72 ± 7.16b	0.006	2.226
MDA (nmol/mL)	14.79 ± 4.97c	8.43 ± 3.28ab	12.47 ± 2.69bc	6.70 ± 1.04a	0.011	1.542

### IFN-γ and TNF-α mRNA expression

**Fig. 1:**
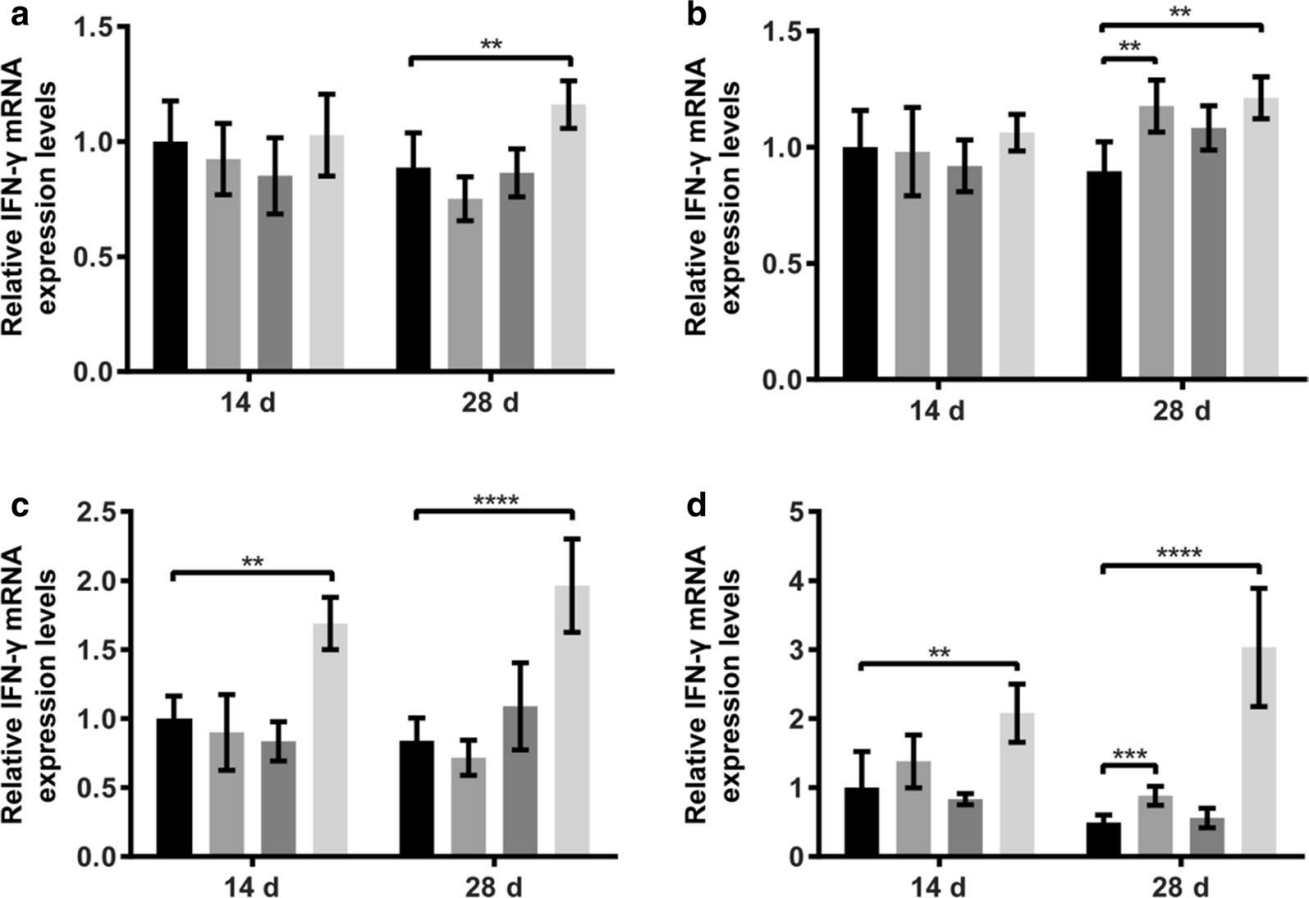
Effects of different dietary supplements on the relative mRNA expression of IFN-γ of laying hens. **a** Expression levels of IFN-γ mRNA in the liver at different time points; **b** Expression levels of IFN-γ mRNA in the spleen at different time points; **c** Expression levels of IFN-γ mRNA in the ileum at different time points; **d** Expression levels of IFN-γ mRNA in the cecum at different time points

**Fig. 2:**
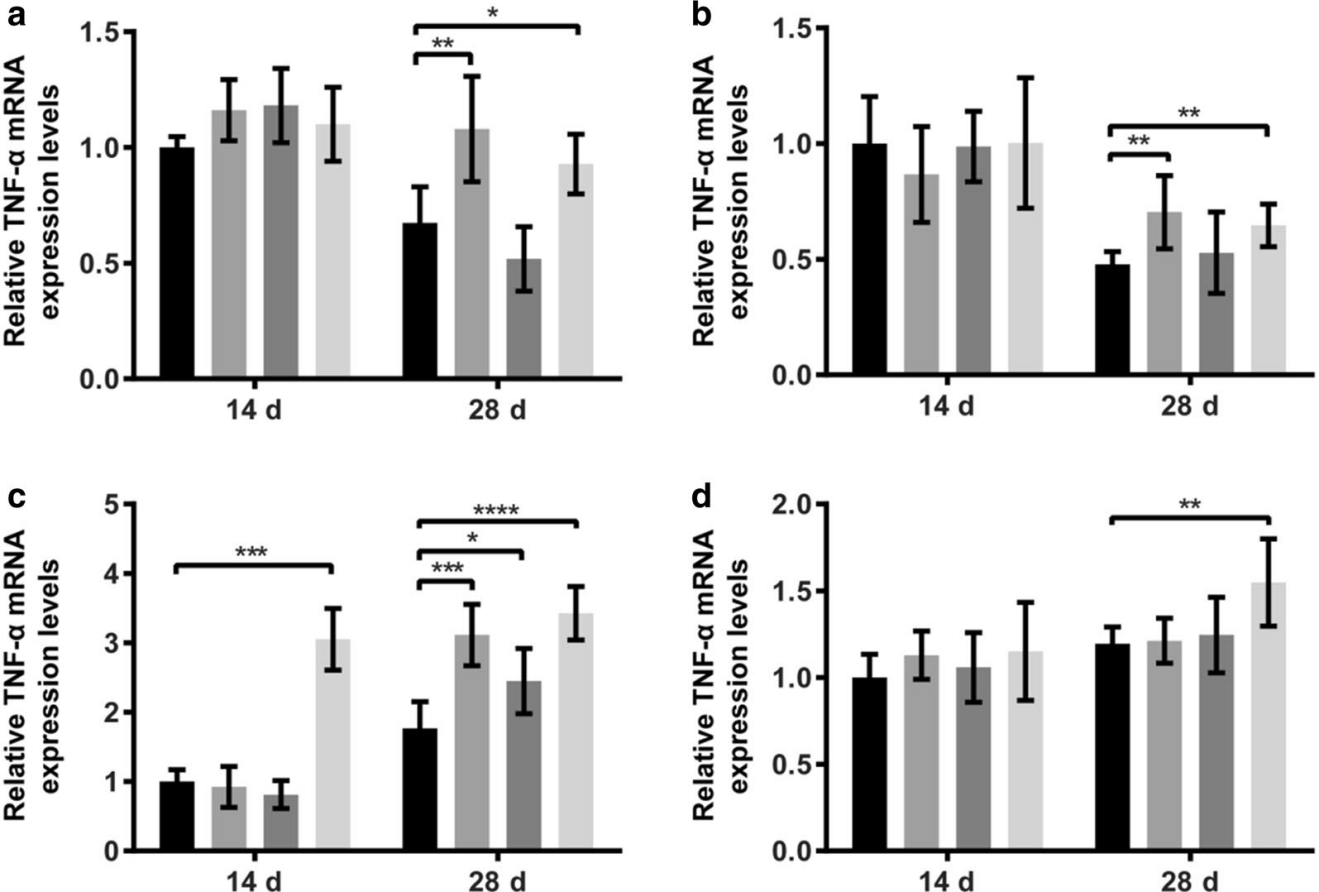
Effects of different dietary supplements on the relative mRNA expression of TNF-α of laying hens. **a** Expression levels of TNF-α mRNA in the liver at different time points; **b** Expression levels of TNF-α mRNA in the spleen at different time points; **c** Expression levels of TNF-α mRNA in the ileum at different time points; **d** Expression levels of TNF-α mRNA in the cecum at different time points

The expression levels of IFN-γ and TNF-α mRNA in the liver, spleen, ileum, and cecum were assessed at 14 and 28 days. As shown in Figs. 1 and 2, the addition of fermented *Astragalus* to the diets respectively increased the mRNA content on day 14 of IFN-γ and TNF-α in the ileum by 1.7-fold (*P* < 0.01) and 3.1-fold (*P* < 0.001), and the mRNA content on day 14 of IFN-γ in the cecum by 2.1-fold (*P* < 0.01). Interestingly, we found that the highest amount of IFN-γ and TNF-α mRNA in the liver, spleen, ileum, and cecum were present in the fermented *Astragalus* group at 28 days.

### Sequencing output

**Fig. 3:**
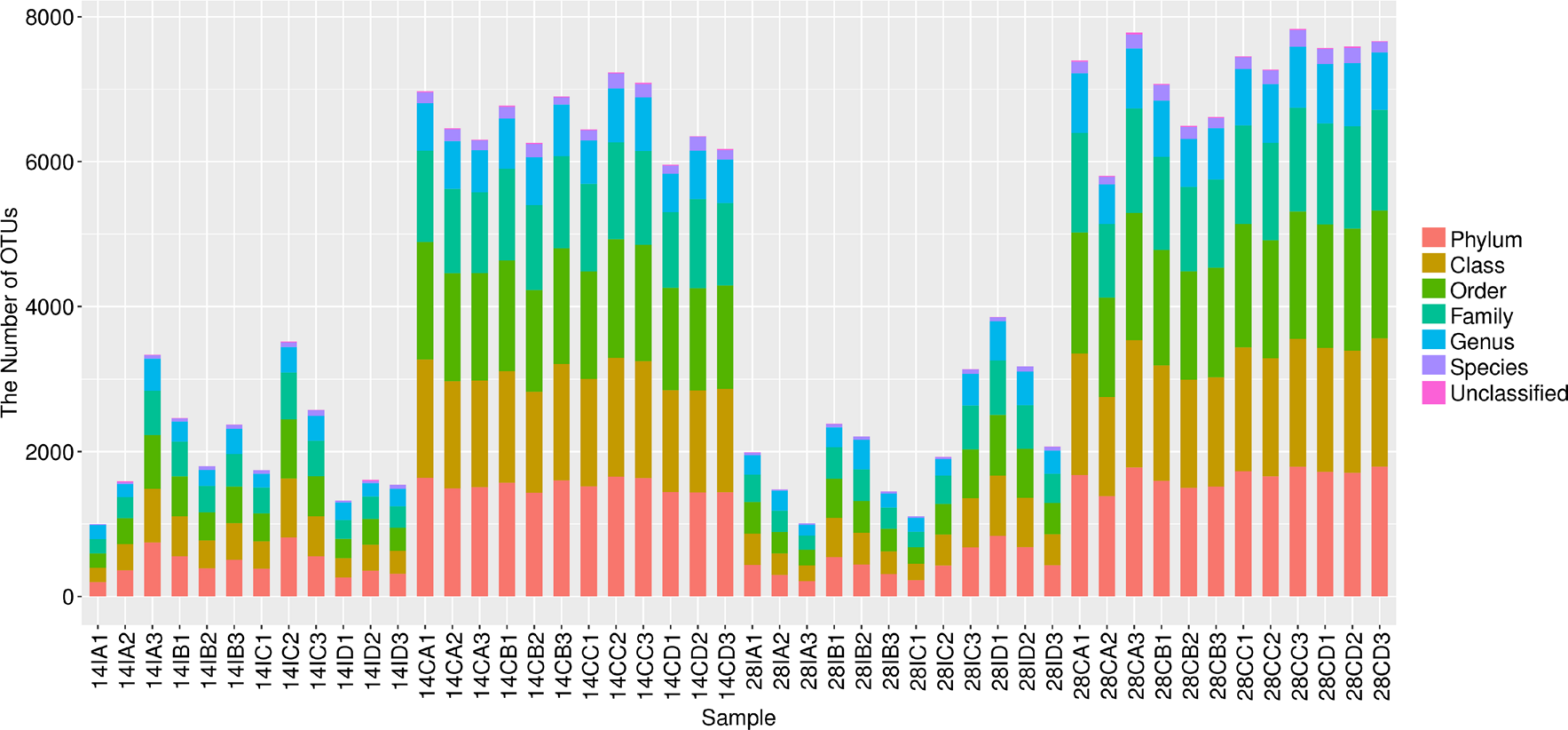
Number of identified taxa (from phyla to species) among the diverse groups

A total of 48 intestinal content samples were analyzed by 16S rRNA gene sequencing and produced a total of 2,006,223 high-quality sequences with an average of 41,796 reads. After OUT clustering at 97% sequence identity, a total of 216,116 OTUs were classified into 49,235 phyla, 48,677 classes, 48,634 orders, 40,101 families, 24,072 genera and 4995 species (Fig. 3).

### Diversity of intestinal microflora

**Fig. 4:**
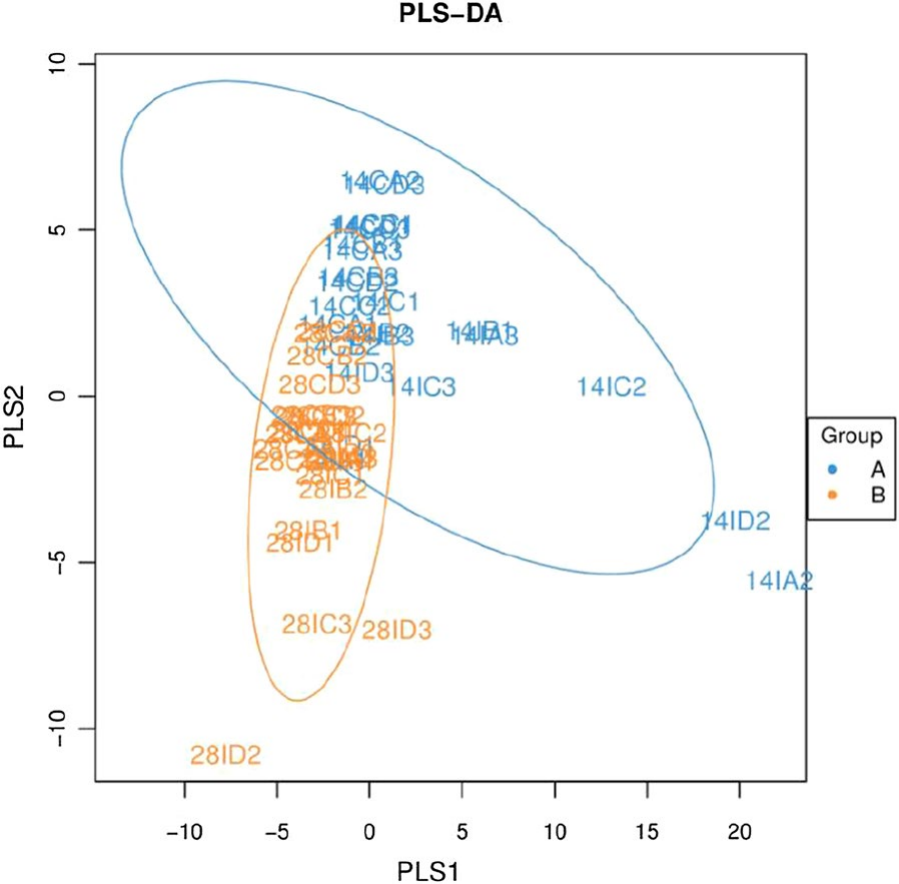
Partial least squares discriminant analysis (PLS-DA) of ileal and cecal microbiota among groups. **a** 14-day sample groups; **b** 28-day sample groups

The α-diversity of ileal and cecal microbiota of four groups on different days are shown in Table 6. For bacteria on day 14, fermented *Astragalus* treatment reduced the Chao1 and ACE index in the cecum in comparison to the control treatment suggesting that fermented *Astragalus* decreased the richness of the bacterial communities. On day 28, the fermented *Astragalus* treatment increased the estimators of diversity (Shannon and Simpson) of the bacterial community in the ileum. PLS-DA was performed to evaluate the similarity (β-diversity) of microbial community structure among groups (Fig. 4). PLS-DA plot defined groups where the samples from different groups occupied distinct positions.

**Table 6:** α-diversity indices of ileum and cecum on days 14 and 28

Parameters	Control	*Astragalus*	*L. plantarum*	Fermented *Astragalus*	*P-*value	SEM
Ileum-14 d						
Simpson	0.84 ± 0.04	0.84 ± 0.09	0.84 ± 0.09	0.79 ± 0.03	0.846	0.045
Chao1	476.76 ± 305.12	526.18 ± 98.32	649.59 ± 233.44	337.49 ± 63.52	0.348	115.944
ACE	493.86 ± 315.21	550.52 ± 106.83	673.67 ± 234.88	346.57 ± 60.90	0.343	118.901
Shannon	3.88 ± 2.14	4.73 ± 0.85	5.28 ± 1.35	3.29 ± 1.17	0.402	0.844
Ileum-28 d						
Simpson	0.60 ± 0.01a	0.55 ± 0.01a	0.90 ± 0.03b	0.91 ± 0.04b	0.000045	0.018
Chao1	349.78 ± 139.33	475.76 ± 144.98	495.18 ± 269.86	722.42 ± 210.22	0.219	114.547
ACE	368.44 ± 146.86	497.43 ± 146.45	527.56 ± 288.28	757.36 ± 217.08	0.222	120.155
Shannon	2.75 ± 1.10a	3.69 ± 0.89ab	4.10 ± 2.10ab	5.79 ± 0.75b	0.110	0.764
Cecum-14 d						
Simpson	0.986 ± 0.006	0.989 ± 0.004	0.990 ± 0.002	0.987 ± 0.003	0.654	0.002
Chao1	1816.94 ± 155.87b	1944.22 ± 132.43b	1923.38 ± 37.30b	1535.10 ± 99.29a	0.009	66.509
ACE	1885.14 ± 164.82b	1926.84 ± 140.76b	1993.92 ± 88.19b	1591.31 ± 143.86a	0.030	79.294
Shannon	8.54 ± 0.15	8.58 ± 0.30	8.67 ± 0.12	8.52 ± 0.24	0.833	0.124
Cecum-28 d						
Simpson	0.99 ± 0.002b	0.98 ± 0.002a	0.99 ± 0.002b	0.99 ± 0.003b	0.026	0.001
Chao1	2008.60 ± 116.80	1910.78 ± 63.71	2008.11 ± 104.08	2022.12 ± 49.39	0.401	47.96
ACE	2158.80 ± 107.42b	1945.56 ± 76.37a	2168.85 ± 123.64b	2168.34 ± 34.51b	0.048	51.71
Shannon	8.73 ± 0.25ab	8.43 ± 0.17a	8.73 ± 0.11ab	8.84 ± 0.18b	0.109	0.106

### Composition of intestinal microflora

**Fig. 5:**
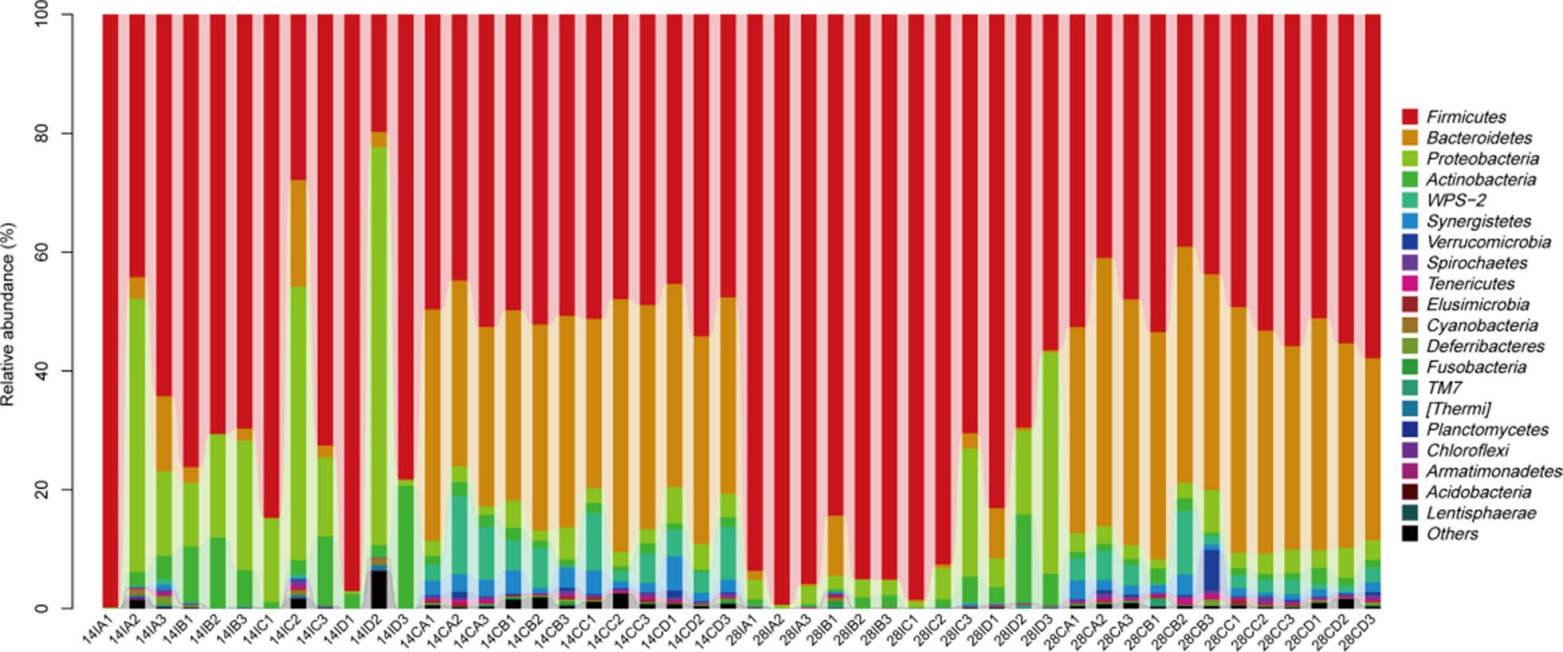
The phylum level distribution of ileal and cecal microbiota among groups. 14IA: 14-d ileum control group; 14IB: 14-d ileum *Astragalus* group; 14IC: 14-d ileum *L. plantarum* group; 14ID: 14-d ileum fermented *Astragalus* group; 14CA: 14-d cecum control group; 14CB: 14-d cecum *Astragalus* group; 14CC: 14-d cecum *L. plantarum* group; 14CD: 14-d cecum fermented *Astragalus* group; 28IA: 28-d ileum control group; 28IB: 28-d ileum *Astragalus* group; 28IC: 28-d ileum *L. plantarum* group; 28ID: 28-d ileum fermented *Astragalus* group; 28CA: 28-d cecum control group; 28CB: 28-d cecum *Astragalus* group; 28CC: 28-d cecum *L. plantarum* group; 28CD: 28-d cecum fermented *Astragalus* group

**Fig. 6:**
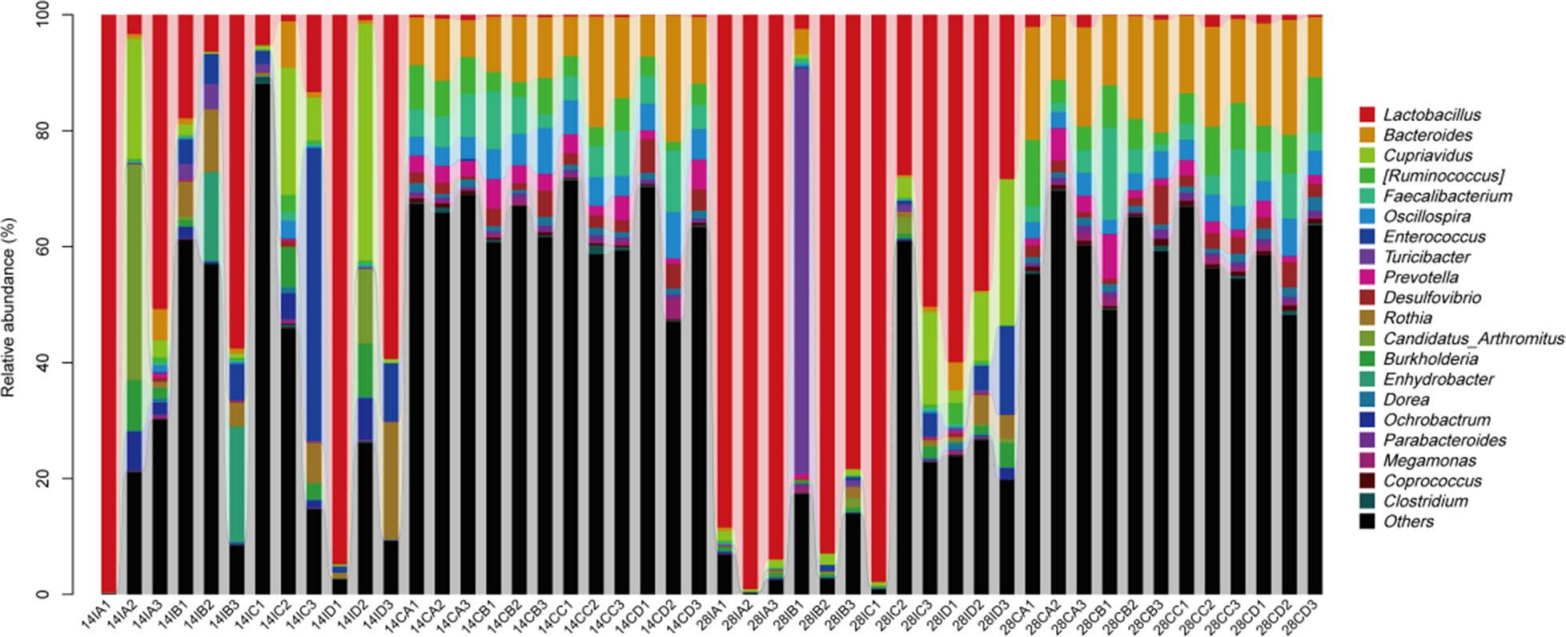
The genus level distribution of ileal and cecal microbiota among groups. 14IA: 14-d ileum control group; 14IB: 14-d ileum *Astragalus* group; 14IC: 14-d ileum *L. plantarum* group; 14ID: 14-d ileum fermented *Astragalus* group; 14CA: 14-d cecum control group; 14CB: 14-d cecum *Astragalus* group; 14CC: 14-d cecum *L. plantarum* group; 14CD: 14-d cecum fermented *Astragalus* group; 28IA: 28-d ileum control group; 28IB: 28-d ileum *Astragalus* group; 28IC: 28-d ileum *L. plantarum* group; 28ID: 28-d ileum fermented *Astragalus* group; 28CA: 28-d cecum control group; 28CB: 28-d cecum *Astragalus* group; 28CC: 28-d cecum *L. plantarum* group; 28CD: 28-d cecum fermented *Astragalus* group

A total of 20 phyla were identified within the intestinal microbiota among 48 samples as shown in Fig. 5. There were three major groups of the intestinal microbiota, including *Firmicutes*, *Bacteroidetes*, and *Proteobacteria*. The relative abundance (%) of cecal bacterial phyla of hens fed with different dietary supplements was presented in approximately the same amount on days 14 and 28. On day 28, fermented *Astragalus* led to a reduced abundance of ileal *Firmicutes*, with an increased abundance of ileal *Bacteroidetes* and *Proteobacteria*. Genus level analysis showed that the *Lactobacillus* and *Bacteroides* accounted for the largest proportion of the intestinal microbiota as shown in Fig. 6. *Lactobacillus* showed high abundance in the ileum and the extremely low abundance in the cecum. In contrast, *Bacteroides* showed high abundance in the cecum and the extremely low abundance in the ileum. On day 14, fermented *Astragalus* addition increased the abundance of cecal *Bacteroides* by 5.06% as compared with the control, with no significant influence on the abundance of ileal *Lactobacillus*. On day 28, fermented *Astragalus* addition significantly decreased the abundance of ileal *Lactobacillus* by 48.51% as compared with the control, with no significant influence on the abundance of cecal *Bacteroides*.

## Discussion

The present study was undertaken to investigate the effects of *L. plantarum* fermented *Astragalus* supplementation on the performance, egg quality, antioxidant status of serum, and gut microbiota in laying hens. When taking out the feeding trial, we observed that diets supplemented with fermented *Astragalus* increased egg production rate (*P* < 0.05) and decreased feed conversion rate (*P* < 0.05), which may likely be attributed to the improvement of laying hen health status. In vivo, free radicals are harmful by-products generated during normal cellular metabolism and are prone to attack unsaturated fatty acid on the biological membrane, triggering lipid oxidation and lipid peroxide accumulation that result in impairment of organism health (Fang et al. 2002). The antioxidant enzymes CAT, GSH-Px, and SOD are associated with free radical scavenging to protect cells from oxidative damage (Zhang et al. 2014). In the present study, supplementation with fermented *Astragalus* resulted in the highest levels of CAT, GSH-Px, SOD, and T-AOC and the lowest level of MDA in the serum (*P* < 0.05) on day 28. Our findings are consistent with our previous studies on broilers (Qiao et al. 2018a), indicating that *Astragalus* fermented by *L. plantarum* can enhance the antioxidant ability of both broilers and laying hens. In our previous research, we compared the differences between fermented *Astragalus* and *Astragalus*. During the fermentation, the pH was markedly reduced, as a consequence of the increase of organic acids content, which could inhibit the growth of miscellaneous bacteria (Qiao et al. 2018c). Fermentation could also elevate the content of *Astragalus* polysaccharide, total saponins, and total flavonoids in *Astragalus*, and fermented *Astragalus* possess more abundant microflora (Qiao et al. 2018c). The increase in the content of *Astragalus* active components by fermentation may be responsible for the enhancement of the performance, egg quality, and antioxidant status of serum in laying hens fed with the basal diet supplemented with 3‰ fermented *Astragalus*.

In recent, *Astragalus* polysaccharide has attracted rising interests in its anti-cancer effects. A previous study has observed that *Astragalus* polysaccharide can significantly enhance the proliferation of spleen lymphocytes and increase phagocytosis of peritoneal macrophages in mice and is capable of up-regulating the expression of IL-2, TNF-α, and IFN-γ in peripheral blood (Li et al. 2020). IFN-γ and TNF-α are cytokines possessing antitumor and immunomodulatory properties and are essential for host immune responses against infection or tissue injury (Li et al. 2019). At the end of our feeding trial (on day 28), *L. plantarum* merely increased the mRNA expression of ileal TNF-α, *Astragalus* increased the mRNA expression of splenic and cecal IFN-γ and that of hepatic, splenic and cecal TNF-α. Interestingly, fermented *Astragalus* significantly increased the mRNA expression of both IFN-γ and TNF-α in all the liver, spleen, ileum, and cecum. However, there are comparatively few findings to date regarding the impact of *L. plantarum* fermented *Astragalus* on host immune responses. We speculate that the increased content of *Astragalus* polysaccharide in the *Astragalus* after fermentation leads to enhance the body’s immune function by increasing the expression of cytokines. Certainly, further investigations will be obliged to fully illustrate that whether there are any endophytic bacteria of *Astragalus* also responsible for immune activation.

Intestinal microbiota plays a major role in maintaining host health, immunity, and production performance, it has become a research hotspot in recent years (Yeoman et al. 2012). In this study, we also evaluated the effect of fermented *Astragalus* on intestinal microbiota of laying hens. Our results showed that fermented *Astragalus* addition increases the diversity of the ileal bacterial community with the increase of feeding time. Furthermore, at the phylum level, *Firmicutes*, *Bacteroidetes,* and *Proteobacteria* were the most dominant phyla in the intestinal microbiota of hens, which are consistent with previous studies (Danzeisen et al. 2011; Yeoman et al. 2012). Interestingly, fermented *Astragalus* addition led to a reduced abundance of ileal *Firmicutes*, with an increased abundance of ileal *Bacteroidetes* and *Proteobacteria*. We speculate that the increased diversity of the ileal bacterial community might be explained by the fact that the abundance of ileal *Firmicutes* was reduced to enhance the abundance of other phyla. At the genus level, *Lactobacillus* as the largest proportion of ileal microbiota of hens is generally highly relevant to feed digestibility (Yan et al. 2017). However, fermented *Astragalus* addition significantly decreased the abundance of ileal *Lactobacillus* by 48.51% as compared with the control at 28 days. These results were totally different from our previous report on the effect of fermented *Astragalus* on the broiler chicken fecal microbiota, which found that the count of *Lactobacillus* was increased in chickens fed fermented *Astragalus* as compared with those in the control group. Those factors responsible for the differences should be further studied.

In conclusion, this study suggested that *L. plantarum* fermented *Astragalus* as an efficient dietary additive could significantly promote the production performance, antioxidant capacity, and ileal microbiota diversity of laying hens during the late laying period. A higher expression level of IFN-γ and TNF-α in the liver, spleen, ileum, and cecum of laying hens supplemented with fermented *Astragalus* indicates a particular role of fermented *Astragalus* on the innate immune system, and this needs a comprehensive investigation in the future to fully illustrate the exact mechanism.

## Data Availability

The datasets generated during this study have been deposited in the National Center for Biotechnology Information sequence read archive (https://www.ncbi.nlm.nih.gov/biosample) under the Accession number SRA: PRJNA533918.

## Abbreviations

*L. plantarum*: *Lactobacillus plantarum*
CAT: Catalase
GSH-Px: Glutathione peroxidase
SOD: Superoxide dismutase
T-AOC: Total antioxidant capacity
MDA: Malondialdehyde
CFU: Colony forming unit
FCR: Feed conversion ratio
ESI: Egg shape index
ESS: Eggshell strength
EST: Eggshell thickness
AH: Albumen height
HU: Haugh unit
YC: Yolk color
YW: Yolk weight
IFN-γ: Interferon gamma
TNF-α: Tumor necrosis factor-alpha
OTUs: Operational Taxonomic Units
PLS-DA: Partial least squares discriminant analysis
